# Baseline Gut Microbiome and Metabolite Profiles Associate with Treatment Response in Breast Cancer Patients Undergoing Neoadjuvant Chemotherapy

**DOI:** 10.3390/diagnostics16030433

**Published:** 2026-02-01

**Authors:** Elza Elizabete Liepina, Elina Sivina, Lelde Jurkane, Zanda Daneberga

**Affiliations:** 1 Institute of Oncology and Molecular Genetics, Riga Stradins University, Pilsonu Street 13, Block 13, LV-1002 Riga, Latvia; 2 Solid Tumor Systemic Treatment Clinic, Riga East University Hospital, Hipokrata Street 4, LV-1079 Riga, Latvia; 3 Clinic of Oncology, Pauls Stradins Clinical University Hospital, Pilsonu Street 13, LV-1002 Riga, Latvia; 4 Department of Biology and Microbiology, Riga Stradins University, Dzirciema Street 16, LV-1007 Riga, Latvia

**Keywords:** breast cancer, gut microbiome, neoadjuvant chemotherapy, metagenomics, metabolomics

## Abstract

**Background/Objectives:** Response to neoadjuvant chemotherapy (NAC) varies substantially among breast cancer patients and is only partially explained by tumor-intrinsic factors. The gut microbiome has emerged as a potential modulator of chemotherapy efficacy, yet its role in breast cancer remains underexplored. This study aimed to characterize gut microbial composition, functional potential, and microbially derived metabolites in breast cancer patients undergoing NAC. **Methods:** baseline stool samples from 39 chemotherapy-naïve breast cancer patients undergoing NAC were analyzed using shotgun metagenomic sequencing and targeted metabolomics. Patients were stratified by pathological complete response (pCR, *n* = 17; no pCR, *n* = 22). Microbial taxonomic and functional profiles, short-chain fatty acids (SCFAs) and bile acids were assessed, with subgroup analysis performed in triple-negative breast cancer (TNBC). **Results:** Patients achieving pCR exhibited significantly higher baseline microbial richness compared to non-responders (*p* = 0.040). Differential abundance analysis revealed enrichment of *Dialister*, *Kineothrix*, and *Jutongia* in responders, whereas *Rothia*, *Leuconostoc*, *Klebsiella*, *Jingyaoa*, *Cuneatibacter*, *Youxingia*, and *Bittarella* were enriched in non-responders. SCFAs (acetate, propionate and butyrate) positively correlated with microbial glucose catabolic pathways, while caproate was negatively associated with multiple amino acid, lipid, vitamin, and cell wall biosynthesis pathways, including peptidoglycan maturation. Metabolomic analysis identified higher deoxycholic acid (DCA) levels in non-responders and increased C6 levels in responders, although these associations did not remain significant after multiple testing correction. Similar trends were observed in the TNBC subgroup (*n* = 15). **Conclusions:** Baseline gut microbiome diversity, taxonomic composition, and functional metabolic potential are associated with response to neoadjuvant chemotherapy in breast cancer, supporting the gut microbiome and its produced metabolites as a potential biomarker of treatment efficacy.

## 1. Introduction

Breast cancer remains the most frequent malignancy among women worldwide and a leading cause of cancer-related mortality, with more than 2.3 million cases and 670,000 deaths reported globally in 2022 [1]. Despite major advances in cancer treatment approaches, with different combinations of multimodality treatments both local and systemic, therapeutic responses remain highly variable among patients. While chemotherapy has been shown to reduce the risk of distant metastases and has an impact on overall breast cancer prognosis and survival, many patients experience dose-limiting hematologic, gastrointestinal and neurological toxicities that not only cause individuals distress, but may also result in treatment modification, discontinuation, and overall reduction in efficacy [2,3]. Identifying biological determinants that predict chemotherapy efficacy and toxicity is therefore crucial for optimizing more individualized treatment strategies and improving patient outcomes. In current clinical practice, NAC efficacy prediction relies predominantly on tumor-intrinsic, tissue-based biomarkers, such as hormone receptor (HR) status, HER2 expression, Ki-67 proliferation index and immune markers, with addition of selected non-immunohistological assays (e.g., HER2 in situ hybridization, germline BRCA testing). However, these markers only partially explain inter-individual variability in treatment response, underscoring the need for additional predictive biomarkers.

In recent years, the gut microbiome has emerged as a promising biomarker and a therapeutic target, representing a new frontier for personalizing chemotherapy and immunotherapy strategies and improving patient outcomes across several malignancies. Microbial communities influence tumor biology and treatment outcomes through diverse mechanisms, including modulation of host immune responses, production of bioactive metabolites (e.g., short-chain fatty acids (SCFAs), secondary bile acids (SBAs)), regulation of systemic inflammation, direct cytotoxic interactions or bacterial translocations, xenobiotic metabolism, and participation in the enterohepatic circulation of hormones such as estrogens [4,5]. SCFAs, including acetate, propionate, and butyrate, are produced by the gut microbiota through fermentation of dietary fiber, they regulate epithelial barrier integrity, modulate regulatory T-cell differentiation, act as histone deacetylase inhibitors and influence cytokine production via G-protein coupled receptors [6]. Through these immunomodulatory and epigenetic mechanisms, SCFAs can alter the tumor microenvironment and potentially modify chemotherapy and immunotherapy sensitivity. Reduced SCFAs production has been linked to increased epithelial permeability, mucosal injury, and bacterial translocation during chemotherapy, exacerbating treatment-induced toxicities [6]. SBAs, including deoxycholic acid (DCA) and litocholic acid (LCA), are produced through microbial transformation of primary bile acids and act as stronger signaling molecules that modulate nuclear receptor activity influencing lipid metabolism, inflammation, and cellular proliferation and apoptosis [7]. Dysregulation in SBA levels has been associated with carcinogenesis and may alter chemotherapy pharmacokinetics via effects on hepatic metabolism and enterohepatic circulation. Furthermore, SBAs contribute to epithelial barrier disruption, further increasing susceptibility to chemotherapy-induced mucosal injury and inflammation [8].

Although microbiome–cancer interactions are well recognized in gastrointestinal, melanoma and lung cancers, their role in breast cancer remains comparatively understudied. Existing evidence indicates that gut microbial composition differs between breast cancer patients and healthy individuals [9]. Preclinical studies using murine models further demonstrate a causal link between microbiome perturbation and chemotherapy response, suggesting that alterations in the gut microbiome may contribute to both therapeutic efficacy and treatment-induced toxicity [10,11]. However, translating these findings into clinical practice has proven challenging. Human studies to date have been limited by methodologic heterogeneity, particularly reliance on 16S rRNS amplicon sequencing, which lacks species- and strain-level taxonomic resolution and provides limited insight into microbial function. With recent advances in sequencing technologies and decreasing costs, shotgun metagenomic sequencing approach has become increasingly accessible, enabling comprehensive characterization of microbial communities and their metabolic capacities. Combining this with complementary quantification of microbially derived metabolites (such as SCFAs and SBAs) provides additional insight into host–microbiome interactions relevant to breast cancer treatment outcomes.

In this study, we integrated shotgun metagenomic profiling with targeted stool metabolomics of SCFAs and SBAs to characterize the gut microbiome in breast cancer patients undergoing neoadjuvant chemotherapy. By examining microbial composition, diversity, metabolic pathways, and metabolite production, our aim was to explore microbial signatures associated with chemotherapy response, thereby contributing to the research of microbiome-based biomarkers and potential therapeutic target development for precision medicine in breast cancer.

## 2. Materials and Methods

### 2.1. Patient Population and Sample Collection

Patients with non-metastatic early breast cancer who had not received any prior systemic therapy were enrolled in this study. All patients were at least 18 years old, had an ECOG performance status of 0 or 1, and provided written informed consent before engaging in the study. The study was carried out in accordance with the Declaration of Helsinki and approved by the Rīga Stradiņš University Research Ethics Committee (No. 2-PĒK-4/492/2025).

Stool samples were collected using home-based Stool Nucleic Acid Collection and Preservation System (Norgen Biotek Corp., Thorold, ON, Canada), ensuring sample homogenization, nucleic acid stabilization, and ambient-temperature transport. Upon receipt, samples were stored at −20 °C for up to one week and subsequently transferred to −80 °C for long-term storage.

### 2.2. Metagenomics Sample Preparation

Microbial DNA was extracted from stool samples using the QIAamp PowerFecal Pro DNA Kit (QIAGEN, Hilden, Germany) according to manufacturer’s instructions. Mechanical homogenization was achieved by using the TissueLyser II instrument (QIAGEN, Hilden, Germany). Isolated DNA’s concentration and purity was assessed using Qubit 4.0 Fluorometer (Invitrogen, Waltham, MA, USA) and NanoDrop 1000 Spectrophotometer (Thermo Fisher Scientific, Waltham, MA, USA). Only samples with A260/280 ratio > 1.8 were selected for next-generation library preparation using the QIAseq FX DNA Library Kit (QIAGEN, Hilden, Germany). Paired-end sequencing (2 × 150 bp) was performed using Illumina’s NextSeq platform(Illumina, San Diego, CA, USA), with a minimum sequencing depth of 25 M reads per sample.

### 2.3. Metagenomics Data Processing and Statistical Analysis

Raw sequencing data were quality-filtered using fastp v1.0.1, removing low-quality reads and sequences shorter than 70 bp. Host-derived reads were removed by mapping against the human reference genome (GRCh38.p13) using bowtie2 v2.5.4. The resulting quality-controlled sequences consisted of average 47 M reads per sample (ranging from 27 M to 78 M). To account for large variability in library size, samples were rarefied to approximately 43 M reads per sample using phyloseq package in Rv1.54.0.

Taxonomical annotation and microbiome relative abundance estimation was performed using MetaPhlAn4 v4.2.4 with a database of ~5.1 M unique clade-specific marker genes (mpa_vJan25_CHOCOPhlAnSGB_202503) [12]. Functional profiling of MetaCyc metabolic pathways were conducted using HUMAnN3 v3.9 with default parameters and normalized to relative abundances [13]. Statistical analyses were performed in R v4.3.0 environment. Alpha diversity was evaluated by calculating observed species count, Shannon and Simpson indices using phyloseq R package v1.54.0. Beta diversity was evaluated using Bray–Curtis dissimilarity index and visualized by Principal Coordinate Analysis (PCoA). Statistical significance of beta diversity differences was determined using Permutational Multivariate Analysis of Variance (PERMANOVA) via ‘adonis2’ from the vegan R package v2.7-2, with 9999 permutations. Alpha diversity metrics, demographic and clinical parameters between therapy outcome groups were compared using Student’s *t*-test, Wilcoxon rank-sum test, chi-squared test or Fisher’s exact test as appropriate, *p* value < 0.05 was considered statistically significant. Continuous variables were assessed for normality using the Shapiro–Wilk test and are represented as mean (standard deviation) or median (range). Categorical variables are presented as frequencies and percentages. Differential abundance testing at species and genus levels was conducted using ANCOM-BC2 with default parameters. Microbial features with relative abundance < 0.01% and that were present in <20% of samples were excluded from testing. A Benjamini–Hochberg multiple testing correction was applied (‘p_adj_method = “BH”’), where *q* < 0.05 was considered to be significant [14]. For microbiome-metabolite exploratory correlation analysis, functional pathway abundances were transformed to relative abundances and filtered by prevalence (<20%) and mean abundance (<0.01%). Pathways labeled as “UNMAPPED” or “UNINTEGRATED” were excluded from downstream analyses. Metabolite concentrations were log_10_ transformed with a pseudocount to account for zero values. Correlations between microbiome pathways and metabolites were computed using Spearman’s rank correlation via the mia R package v.1.18.0. Both raw *p*-values and Benjamini–Hochberg adjusted *p*-values were calculated to account for multiple testing.

### 2.4. Targeted Metabolomics Sample Preparation

Targeted metabolomics in all fecal samples was performed to measure: (1) SCFAs: acetic acid (C2), propionic acid (C3), isobutyric acid (iC4), butyric acid (C4), isovaleric acid (iC5), valeric acid (C5), caproic acid (C6), and isocaproic acid (iC6); (2) bile acids: cholic acid (CA), chenodeoxycholic acid (CDCA), deoxycholic acid (DCA), glycocholic acid (GCA), glycochenodeoxycholic acid (GCDCA), glycodeoxycholic acid (GDCA), glycolithocholic acid (GLCA), litocholic acid (LCA), muricholic acid (MCA), taurocholic acid (TCA), taurochenodeoxycholic acid (TCDCA), taurodeoxycholic acid (TDCA), taurolitocholic acid (TLCA), tauroursodeoxycholic acid (TUDCA), and ursodeoxycholic acid (UDCA).

For targeted bile acid analysis, 0.200 g of thawed fecal sample was mixed with 20 µL of an isotopically labeled internal standard mixture (MSK-BA1-1 Bile Acid Standard Mix 1—Unconjugated, Cambridge Isotope Laboratories; MSK-A2-1.2) and 1 mL of methanol. The mixture was thoroughly vortexed and centrifuged. After centrifugation, 100 µL of the supernatant was collected for bile acid measurements.

For short-chain fatty acid (SCFA) analysis, a separate aliquot of 0.200 g of thawed fecal sample was mixed with 200 µL of SCFA internal standard solution, 250 µL of 50% (*w*/*v*) sulfuric acid, and 1 mL of methyl tert-butyl ether (MTBE). The samples were vortexed and centrifuged, after which 500 µL of the supernatant was collected for GC-FID analysis.

#### 2.4.1. SCFA Measurements with GC-MS

SCFA quantification was performed using an Agilent Technologies 6890 gas chromatograph equipped with a flame ionization detector (FID) (Agilent Technologies Inc., Santa Clara, CA, USA). Separation was achieved on a DB-FFAP capillary column (30 m × 0.25 mm × 0.25 µm), with helium used as the carrier gas. The system was operated in constant flow mode at 2 mL/min.

Samples (2 µL) were injected using an Agilent Technologies 7683B autosampler (Agilent Technologies Inc., Santa Clara, CA, USA) in split mode (split ratio 1:5; split flow 10 mL/min). The inlet temperature was maintained at 250 °C using an Agilent 5183-4647 split/splitless liner (Agilent Technologies Inc., Santa Clara, CA, USA). The oven temperature program was as follows: initial temperature of 100 °C, ramped to 170 °C at 20 °C/min, followed by a second ramp to 225 °C at 2 °C/min, and a final ramp to 245 °C at 20 °C/min, with an 8 min hold. The total run time was 40 min per injection.

The FID temperature was set to 250 °C, with air and helium make-up gas flow rates of 450 mL/min and 50 mL/min, respectively. Data were acquired at a 50 Hz scan rate. Raw data processing was performed using MSD ChemStation D.03.00.611 (Agilent Technologies Inc., Santa Clara, CA, USA). Calibration curves were generated using standard solutions of acetic acid, propionic acid, butyric acid, isobutyric acid, pentanoic acid, and isopentanoic acid (Thermo Fisher Scientific, Waltham, MA, USA), prepared in the concentration range of 1–15,000 µM. Calibration was based on the analyte-to-internal standard peak area ratio and was fitted using linear regression. Automated peak integration and calibration were manually inspected and corrected where necessary. Final results were exported to Excel for further statistical analysis.

#### 2.4.2. Bile Acid Measurements with UHPLC-MS

Bile acid analysis was performed using a Vanquish Core UHPLC system (Thermo Fisher Scientific, Waltham, MA, USA) coupled to an Orbitrap Exploris 120 mass spectrometer (Thermo Fisher Scientific, Waltham, MA, USA). Chromatographic separation was achieved on a ZORBAX Eclipse XDB-C18 analytical column (1.8 µm, 2.1 × 50 mm; Agilent Technologies Inc., Santa Clara, CA, USA), equipped with a BEH C18 VanGuard pre-column (2.1 × 5 mm; Waters, Milford, MA, USA). The column temperature was maintained at 40 °C, and the injection volume was 2 µL. The mobile phase consisted of 0.1% (*v*/*v*) formic acid in water (phase A) and 0.1% (*v*/*v*) formic acid in acetonitrile (phase B). Gradient elution was performed at a flow rate of 0.4 mL/min, with a total run time of 10 min.

Mass spectrometric detection was carried out in negative electrospray ionization (ESI) mode with a spray voltage of 2.5 kV. The scan range was set from *m*/*z* 50 to 600, with a mass resolution of 60,000. The gas heater temperature was 400 °C, the capillary temperature was 350 °C, the auxiliary gas flow rate was 12 arbitrary units, and the nebulizing gas flow rate was 50 arbitrary units. Quantification was performed using seven-point calibration curves with internal standardization. LC-MS data processing and quantification were carried out using TraceFinder 5.1 General Quan software (Thermo Fisher Scientific, Waltham, MA, USA). All reported bile acids were identified at Level A, based on matching retention time and mass spectral data to authentic reference standards previously validated on the analytical system.

### 2.5. Metabolomics Data Processing and Statistical Analysis

Statistical analyses and visualizations were performed using MetaboAnalyst 6.0 [15].

All quantified metabolite concentrations (μM) were normalized using log transformation followed by Pareto scaling. Missing values were imputed with a pseudocount with a value of 0.001. Linear modeling was performed in MetaboAnalyst 6.0 using the limma framework, to assess differences between treatment response, with HR status incorporated into the model matrix as an adjusting variable.

## 3. Results

### 3.1. Patient Characteristics and Clinical Outcome

In total, 40 newly diagnosed chemotherapy-naive breast cancer patients that underwent neoadjuvant chemotherapy (NAC) were included in this study. One patient was excluded after antibiotic use, leaving 39 patients in the final cohort. Patients were stratified according to treatment outcome, defined as achievement of pathological complete response (pCR). Attainment of pCR after NAC is considered a prognostic factor for event-free survival (EFS) and overall survival (OS), particularly in HER2 positive and triple negative breast cancer (TNBC) [16]. In our cohort, 17 of 39 patients (43.6%) achieved pCR. The distribution of molecular subtypes and therapy regimens are presented in Table 1. pCR was predominantly observed in patients with HER2 positive and TNBC. Among HER2 positive tumors, pCR occurred mainly in patients receiving taxane-platinum-targeted therapy, whereas no pCR was observed in those treated with anthracycline-based regimens combined with targeted therapy. In Luminal A cancer, pCR was limited to patients treated with taxane-platinum-targeted therapy. No pCR was observed in patients with Luminal B (HER2 negative) tumors, regardless of treatment regimen. In TNBC, pCR rates varied by regimen, with responses observed across multiple anthracycline- and taxane-based combinations, including those incorporating platinum and/or immunotherapy.

Clinical characteristics of the study group are summarized in Table 2. Although the two treatment outcome groups were heterogeneous, they were balanced, apart from statistically significant differences in germline pathogenic variant (PV) status of hereditary breast and ovarian cancer syndrome (HBOC). Median age did not differ significantly between responders (pCR) and non-responders (no-pCR) (50.0 [34.0–65.0] vs. 52.5 [30.0–80.0] years, Mann–Whitney *U* = 168, *p* = 0.600, *r* = −0.102). Similarly, mean body surface area (bsa) (1.87 ± 0.15 vs. 1.84 ± 0.18 m^2^, Student’s *t*-test *t(37)* = 0.579, *p* = 0.566, *d* = −0.187) and clinical stage at diagnosis did not differ significantly between groups (Mann–Whitney *U* = 154, *p* = 0.810, *r* = −0.046), with most patients presenting with stage II (41.7%) or stage III (41.7%) breast cancer. Hormone receptor (HR) status showed a trend toward association with pCR, with a higher proportion of HR-negative tumors in the pCR group (Fisher’s test *p* = 0.056). Tumor grade demonstrated a non-significant trend, with grade 3 tumors being more frequent among patients achieving pCR (chi-square test *x^2^(1)* = 2.84, *p* = 0.092, *Cramer’s V* = 0.270), reflecting higher responsiveness of aggressive tumors to cytotoxic therapy. Notably, germline pathogenic variants were detected exclusively in patients who achieved pCR (5/17, 12.8%), whereas none were observed in the no-pCR group (0/22), representing a statistically significant association (Fisher’s test *p* = 0.011).

### 3.2. Gut Microbiome Diversity and Taxonomic Associations with Pathological Response

**Figure 1:**
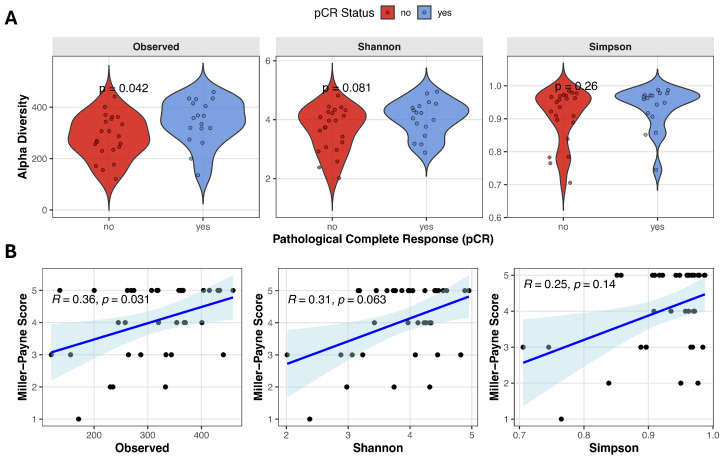
Gut microbiome alpha diversity and correlation with pathological response. (**A**) Violin plots comparing alpha diversity scores (Observed species count, Shannon and Simpson indices) between pCR and no-pCR groups. (**B**) Spearman correlation analysis between Miller–Payne grade and alpha diversity metrics. Blue line with blue shadow represents the fitted trend line with the 95% confidence interval, black points represent each individual sample.

Alpha diversity reflects the microbial diversity within an individual stool sample and can be quantified using various metrics that capture richness and/or evenness. We evaluated the relationship between baseline alpha diversity and pathological complete response, using three alpha diversity measures derived from metagenomic data: Observed species, Shannon diversity, and Simpson index. Observed species count was significantly higher in the pCR group compared with no-pCR patients (343.0 ± 88.2 vs. 284.1 ± 83.5, Student’s *t*-test *t(37)* = −2.13, *p* = 0.040, *d* = −0.688), and Shannon diversity showed a trend toward higher values in responders (4.06 ± 0.61 vs. 3.67 ± 0.75, Student’s *t*-test *t(37)* = −1.74, *p* = 0.089, *d* = −0.563). Simpson index was slightly higher in patients achieving remission, although it did not reach statistical significance (0.96 [0.74–0.99] vs. 0.94 [0.71–0.98], Mann–Whitney test *U* = 144, *p* = 0.229, *r* = 0.230) (Figure 1A). Spearman correlation analysis revealed a moderate positive correlation between observed species and Miller–Payne grade (Observed species: *ρ* = 0.359, *p* = 0.031), suggesting that higher microbial richness is associated with better pathological response (Figure 1B). A positive but non-significant trend was observed between Miller–Payne grade and Shannon diversity (*ρ* = 0.314, *p* = 0.063), while no significant association was detected with Simpson index (*ρ* = 0.250, *p* = 0.141).

**Figure 2:**
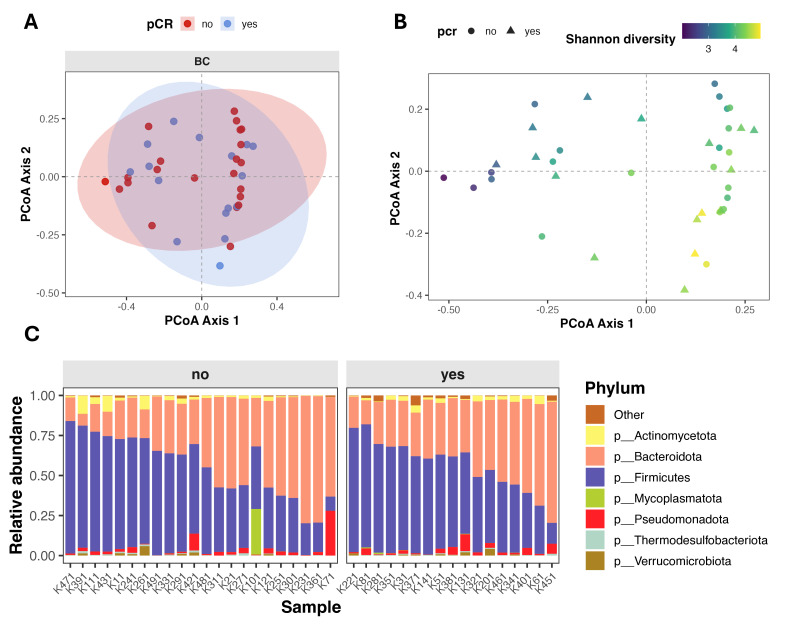
Gut microbiome composition and beta diversity in breast cancer patients stratified by pathological complete response (pCR). (**A**,**B**) Principal Coordinates Analysis (PCoA) of beta diversity, showing sample clustering based on microbial composition. Points are colored by pCR status (**A**) and by Shannon index (**B**). (**C**) Stacked bar plot showing relative abundance of top seven microbial phyla, faceted by pCR group.

Beta diversity (intersample diversity) was assessed using Bray–Curtis distance metric, that captures differences in microbial community composition based on relative abundance and presence/absence of taxa. Principal coordinate analysis (PCoA) showed substantial overlap between patients with different treatment outcomes (Figure 2A), and permutational multivariate analysis of variance (PERMANOVA) revealed no significant differences in overall microbial community composition (Bray–Curtis: *F* = 0.895, *R^2^* = 0.024, *p* = 0.568; Jaccard: *F* = 0.943, *R^2^* = 0.025, *p* = 0.548). Notably, partial clustering of samples appeared to be driven by differences in alpha diversity rather than pCR status (Figure 2B).

**Figure 3:**
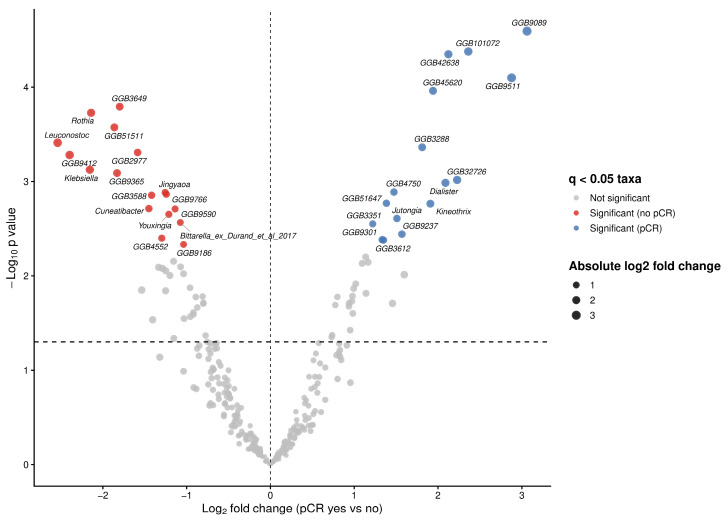
Differentially abundant microbial taxa at genus level associated with pathological complete response (pCR) status. Volcano plot showing differential abundance of microbial genera between patients achieving pCR and those without pCR. Log fold changes (LFC) estimated using ANCOM-BC2 are plotted against −log10 adjusted *p*-values following Benjamini–Hochberg correction. Vertical dashed line indicates no change (LFC = 0), while horizontal dashed line denoted the nominal significance threshold (*p* = 0.005).

To investigate associations between bacterial taxa and therapy outcomes in our cohort, we performed differential abundance analysis using ANCOM-BC2 at genus level. We identified multiple taxa exhibiting large effect sizes (log fold change (LFC) > 2) and statistically significant differences (*q* < 0.05) between both therapy outcome groups (Figure 3). Significant increases in the abundances of *Dialister* (*W* = 4.57, LFC = 2.09, *q* = 0.021), *Kineothrix* (*W* = 3.68, LFC = 1.91, *q* = 0.025), *Jutongia* (*W* = 4.61, LFC = 1.51, *q* = 0.030) genera, as well as assembled genomes were observed in patients achieving pCR (Figure 3; Table 3). Conversly, taxa significantly enriched in non-pCR patients included *Rothia* (*W* = −8.12, LFC = −2.14, *q* = 0.009), *Leuconostoc* (*W* = −6.34, LFC = −2.54, *q* = 0.014), *Klebsiella* (*W* = −6.29, LFC = −2.16, *q* = 0.018), *Jingyaoa* (*W* = −4.41, LFC = −1.26, *q* = 0.022), *Cuneatibacter* (*W* = −5.24, LFC = −1.45, *q* = 0.026), *Youxingia* (*W* = −3.64, LFC = −1.21, *q* = 0.028), *Bittarella* (*W* = −3.76, LFC = −1.08, *q* = 0.032) genera and assembled genomes (Figure 3; Table 3).

### 3.3. Metabolomic Profiling and Functional Metabolite–Microbiome Correlations with Treatment Response

**Figure 4:**
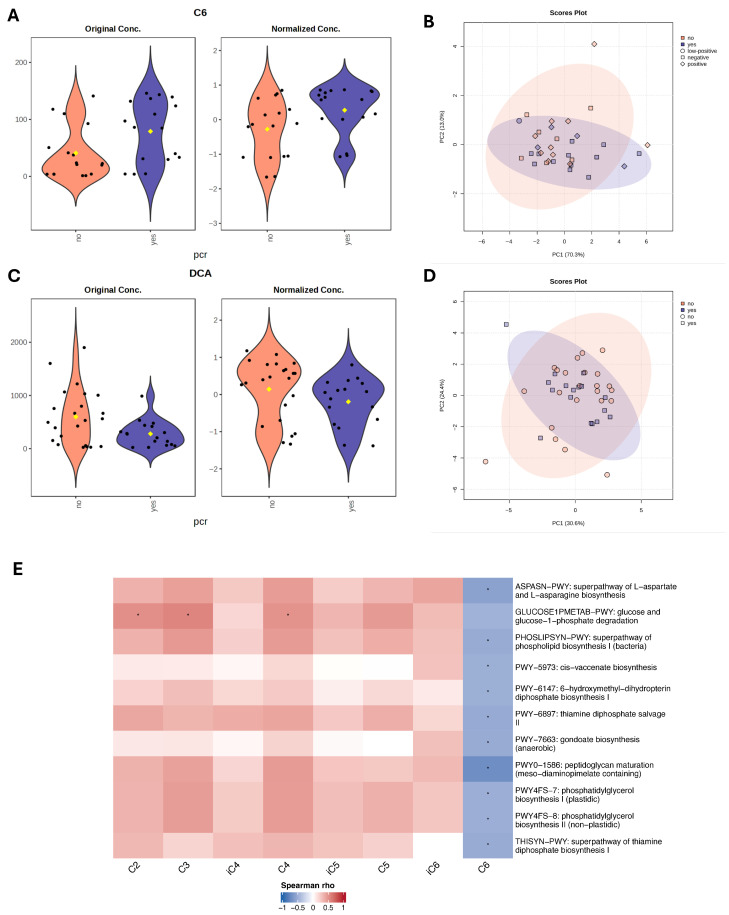
Metabolite differences associated with pathological complete response (pCR) status. (**A**,**C**) Violin plots with nominally significant (*p* < 0.05) metabolites identified by linear regression model (based on limma) for therapy response groups with HR status as a covariate. Black dots represent individual samples, yellow dot indicates the group mean. (**B**,**D**) PCA plots based on Euclidean distance distributions for SCFAs (*n* = 34) (PERMANOVA F-value: 1.2329; R-squared: 0.0371; *p*-value (based on 999 permutations): 0.29) (**B**) and SBAs (*n* = 39) (PERMANOVA F-value: 0.035731; R-squared: 0.0009394; *p*-value (based on 999 permutations): 0.977) (**D**). (**E**) Heatmap showing Spearman correlations between SCFAs and microbial metabolic pathways. Positive correlations are indicated by red, negative by blue. Only pathways with >20% prevalence and mean relative abundance > 0.001% are shown. * indicates false discovery rate (FDR) adjusted *p* < 0.2.

Next, we performed metabolomic analysis to identify individual metabolites that differentiate responders and non-responders to NAC. For bile acid analysis, all study cohort patients (*n* = 39) were included, however for SCFA analysis, five samples were excluded due to poor quality, leaving 34 samples for statistical evaluation. Using linear regression analysis, controlling for hormone receptor (HR) status, we identified higher DCA (LFC = −0.48, *t* = −2.07, *p* = 0.04, *adj.p* = 0.59) in non-responders, and a trend of GDCA (LFC −0.43, *t* = −1.89, *p* = 0.06, *adj.p* = 0.59) higher in non-responders. C6 was higher (LFC = 0.57, *t* = 2.28, *p* = 0.02, *adj.p* = 0.34) in responders. Although these metabolites showed nominal significance, none remained statistically significant after adjustment for multiple testing (Figure 4A,C).

Spearman correlation analysis was performed to assess associations between microbiome metabolic pathways and short-chain fatty acids. Acetate (C2), propionate (C3), and butyrate (C4) revealed positive correlations with the glucose and glucose-1-phosphate degradation pathway (GLUCOSE1PMETAB-PWY). The strongest trend was observed for C3 (*ϱ* = 0.57, *p* = 0.001, FDR = 0.17), followed by C2 (*ϱ* = 0.52, *p* = 0.002, FDR = 0.20) and C4 (*ϱ* = 0.51, *p* = 0.002, FDR = 0.20), suggesting that higher SCFA levels were associated with increased microbial glucose catabolic activity. In contrast, caproate (C6) was negatively correlated with multiple microbial pathways, including amino acid biosynthesis (ASPASN-PWY, *ϱ* = −0.58, *p* < 0.001, FDR = 0.17), lipid metabolism (PHOSLIPSYN-PWY, *ϱ* = −0.50, *p* = 0.003, FDR = 0.20), cis-vaccenate biosynthesis PWY-5973 (*ϱ* = −0.53, *p* = 0.002, FDR = 0.20), PWY-7663 (*ϱ* = −0.50, *p* = 0.003, FDR = 0.20). Additionally, negative trends were observed with pathways involved in cofactor and vitamin metabolism, including PWY-6147 (tetrahydrofolate precursor biosynthesis), PWY-6897 (thiamine diphosphate salvage II), and THISYN-PWY (thiamine diphosphate biosynthesis I). However, none of these associations remained statistically significant, after multiple testing adjustment. The strongest negative association was detected between C6 and PWY0-1586 (peptidoglycan maturation, meso-diaminopimelate-containing) (*ϱ* = −0.72, *p* < 0.001, FDR = 0.004) (Figure 4E; Table 4). Correlation analysis was also performed between microbial functional pathways and bile acid metabolites, with results presented in Appendix A Table A1.

### 3.4. Association of Baseline Gut Microbiota Composition and Clinical Benefit in TNBC Sub-Cohort

To evaluate whether the observed associations in the full cohort remained consistent after accounting for the potential confounding effect of molecular subtype, we examined the gut microbiota in a smaller, but more homogeneous sub-cohort consisting only of TNBC patients (*n* = 15). Within this group, nine patients achieved pathological complete response, while six patients did not. Study group characteristics are summarized in Table 5.

**Figure 5:**
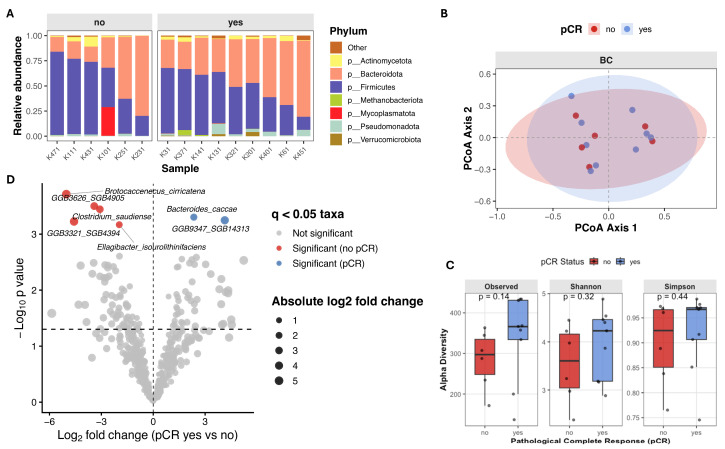
Gut microbiome composition and diversity in TNBC patients stratified by pathological complete response (pCR) (**A**) Stacked bar plot showing relative abundance of top seven microbial phyla, faceted by pCR group. (**B**) Principal Coordinates Analysis (PCoA) of beta diversity, showing sample clustering based on microbial composition. (**C**) Boxplots of alpha diversity scores (Observed species count, Shannon and Simpson indices) in pCR and no-pCR groups. (**D**) Volcano plot from ANCOM-BC2 showing compositional differences in microbial species between TNBC patients with pCR (*n* = 9) versus no-pCR (*n* = 6). Taxa with Benjamini–Hochberg adjusted *q* < 0.05 were considered significant. Each point represents species, with point color indicating the significant taxa magnitude and direction of effect size. Taxa enriched in pCR group are colored in blue, and taxa enriched in patients without clinical benefit are colored red.

Alpha diversity analysis revealed higher microbial richness and diversity in patients who achieved pCR, although these differences did not reach statistical significance. Observed species count (median 366.0 [136.0–434.0] vs. 297.0 [171.0–363.0], Mann–Whitney *U* = 14, *p* = 0.140, *r* = 0.481), Shannon index (4.23 [2.88–4.89] vs. 3.61 [2.38–4.45], Mann–Whitney *U* = 18, *p* = 0.316, *r* = 0.333) and Simpson index (0.97 [0.75–0.99] vs. 0.92 [0.77–0.97], Mann–Whitney *U* = 20, *p* = 0.444, *r* = 0.259) were higher in patients who achieved pCR (Figure 5C). Similarly, in TNBC patients principal coordinate analysis (PCoA) based on beta-diversity metrics showed substantial overlap between patients with different treatment outcomes, and PERMANOVA revealed no significant differences in overall microbial community composition (Bray–Curtis: *F* = 0.775, *R^2^* = 0.056, *p* = 0.776; Jaccard: *F* = 0.862, *R^2^* = 0.062, *p* = 0.758) (Figure 5B). Differential abundance analysis using ANCOM-BC2 revealed several taxa enriched in non-responders: *Brotocaccenecus cirricatena* (*W* = −13.37, LFC = −5.01, *q* = 0.049), *Clostridium saudiense* (*W* = −11.49, LFC = −3.08, *q* = 0.049), *Ellagibacter isourolithinifaciens* (*W* = −5.51, LFC = −1.98, *q* = 0.049), and GGB3321 SGB4394 (*W* = −7.80, LFC = −4.61, *q* = 0.049), and GGB3626 SGB4905 (*W* = −11.86, LFC = −3.42, *q* = 0.049) assembled genomes. While *Bacteroides caccae* (*W* = 5.70, LFC = 2.34, *q* = 0.049) and GGB9347 SGB14313 (*W* = 10.09, LFC = 4.12, *q* = 0.049) was observed more enriched in responders (Figure 5D; Table 6).

**Figure 6:**
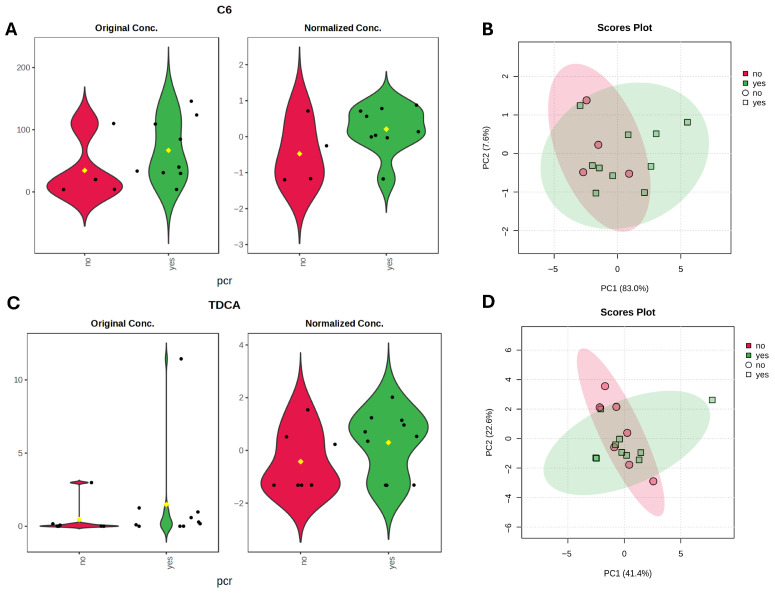
Metabolite differences associated with pathological complete response (pCR) status in TNBC subgroup. (**A**,**C**) Violin plots with metabolites with largest effect sizes (LFC) identified by linear regression model (based on limma) for TNBC therapy response groups. Black dots represent individual samples, yellow dot indicates the group mean. (**B**,**D**) PCA plots based on Euclidean distance distributions for SCFAs (PERMANOVA F-value: 1.6942; R-squared: 0.12372; *p*-value (based on 999 permutations): 0.196) (**B**) and SBAs (PERMANOVA F-value: 0.33667; R-squared: 0.021952; *p*-value (based on 999 permutations): 0.81) (**D**).

SCFA metabolite analysis using linear regression models, identified caproate (C6) as the metabolite with the largest effect size enriched in the pCR group (LFC = 0.69, *t* = 1.65, *p* = 0.10, *q* = 0.48). Bile acid analysis identified taurodeoxycholic acid (TDCA) (LFC = 0.72, *t* = 1.54, *p* = 0.12, *q* = 0.96) as elevated in responders, although neither metabolite reached statistical significance. The C6 elevation is in line with the overall cohorts’ results, whereas TDCA, a conjugate of DCA, contrasts with the trend observed for DCA, which was elevated in non-responders. Examination of the DCA/TDCA ratio revealed a trend toward a higher ratio in non-responders (median 8597 [8–107070] vs. 1439 [5–19965], Mann–Whitney *U* = 11, *p* = 0.068, *r* = −0.593) (Figure 6).

## 4. Discussion

To the best of our knowledge this is one of the few studies to investigate the association between gut microbiome composition, microbially derived metabolites, and pathological complete response (pCR) to neoadjuvant chemotherapy (NAC) in breast cancer patients using shotgun metagenomic and targeted metabolomic approach.

In this cohort of chemotherapy-naive breast cancer patients treated with NAC, a pCR rate of 43.6% was observed. As expected, pCR occurred predominantly in HER2-positive and TNBC tumors, supporting the known chemosensitivity of these subtypes. Baseline clinical and demographic characteristics were comparable between responders and non-responders, indicating minimal confounding by age, body surface area, or clinical stage. However, trends toward higher pCR rates in hormone receptor-negative and grade 3 tumors were observed, consistent with prior evidence of HR-negative breast cancer chemosensitivity, which is long known to achieve higher pCR rates following neoadjuvant chemotherapy [17]. Importantly, NAC regimens varied substantially across molecular subtypes in our cohort, in accordance with current treatment standards. This treatment heterogeneity represents another potential confounder beyond HR status alone and may have contributed to the observed differences in both observed microbial composition and metabolomic profiles and actual clinical treatment outcomes. This factor should therefore be carefully considered in the interpretation of our findings and addressed in future studies with more homogeneous distribution of treatment strategies.

Interestingly, all patients carrying inherited pathogenic variants were observed among responders, while emerging evidence suggests an association between *BRCA1/2* PV and NAC response, the small number of affected patients and heterogeneity of chemotherapy regimens in our cohort preclude definitive conclusions [18,19].

Our results demonstrate that higher gut microbial alpha diversity, specific bacterial taxa, and distinct metabolites are associated with favorable treatment outcomes. However, our findings must be interpreted in the context of substantial limitations related to sample size and heterogeneity, which constrain the generalizability and clinical applicability of results. Although several associations exhibited large effect sizes, suggesting potential biological relevance, most did not retain significance after rigorous correction for multiple testing. Importantly, some of these signals were partially preserved when explored in a more homogeneous TNBC sub-cohort. However, given the very limited sample size, these results should be considered exploratory, warranting strong validation in a larger cohort.

Patients achieving pCR exhibited significantly higher microbial richness, as measured by observed species counts, with concordant trends in Shannon and Simpson diversity indices. Alpha diversity also demonstrated a moderate positive correlation with Miller–Payne grade, suggesting that higher baseline gut microbiome diversity is associated with greater tumor regression following NAC. These results are in line with some previous studies, where alpha diversity was correlated with both prolonged progression-free survival (PFS) and overall survival (OS), as well as response to neoadjuvant chemotherapy [20,21]. While other studies have reported conflicting results—showing no association with clinical benefit to NAT [22].

At the taxonomic level, we observed significant differences between the two treatment outcome groups. Genera enriched in patients achieving pCR included known or putative SCFA producers and gut commensals. *Dialister* and its representative species *D. invisus* is associated with propionate and acetate production through the succinate pathway [23]. However, it has also been found in higher abundances in cancer patients, although most evidence remains correlative and their specific role in cancer outcomes requires further investigation [24]. *Kineothrix* (*SGB4886*) and *Jutongia* (*J. hominis*) are genera within the *Lachnospiraceae* family, whose members are also known SCFA producers [25]. While both genera remain poorly characterized in the context of cancer, higher *Kineothrix* abundance has been associated in murine models with improved lipid profiles, lower inflammatory markers, and better intestinal barrier function [26].

Conversely, genera enriched in non-responders included *Rothia*, *Klebsiella*, *Lactococcus* and less characterized genera such as *Jingyaoa*, *Cuneatibacter*, *Youxingia*, *Bittarella*. In our study, *Rothia* genus was represented only by *Rothia mucilaginosa*; a facultative anaerobic bacteria commonly found in the human oral cavity and upper respiratory tract, it has also been detected in fecal microbiome profiles [27]. Its metabolic activity primarily involves fermentation of carbohydrates to produce lactic acid [28]. *R. mucilaginosa* can also occasionally act as an opportunistic pathogen, particularly in immunocompromised individuals [29]. *Klebsiella* (*K. pneumoniae*), also an opportunistic pathogen, has been observed as a microbial marker enriched in the gut microbiota of postmenopausal breast cancer patients compared with controls [30]. In our study, *Lactococcus* genera was represented by two species, *L. cremoris* and *L. lactis*. These bacteria are generally regarded as safe and are widely used in food production and fermentation. While some cancer studies have reported correlations with disease risk, the mechanistic links underlying these associations remain unclear [31,32].

When exploring microbial composition differences in TNBC patients, higher abundance of *Bacteroides caccae* was observed in patients achieving pCR. Members of *Bacteroides* genus are known SCFAs producers and have been linked with improved responses to immune-checkpoint blockade in melanoma cancer patients [33]. In contrast, patients without pCR exhibited higher relative abundances of *Ellagibacter isourolithinifaciens*, *Clostridium saudiense*, and *Brotocaccenecus cirricatena*. *E. isourolithinifaciens* participates in the metabolism of dietary ellagic acid to isourolithin metabolites, while members of *Clostridium* are known to participate in secondary bile acid metabolism and to influence intestinal barrier integrity and inflammation, their roles on treatment outcomes remains unclear [34].

Metabolite analysis indicated higher levels of caproic acid (C6) in responders. Although less studied than acetate, propionate and butyrate, caproic acid also is a microbial fermentation product derived from fiber and amino acids. In contrast, higher deoxycholic acid (DCA) levels were observed in non-responders, which is in line with the literature. DCA and LCA have been implicated in pathological states, including promotion of inflammation and cancer-promoting processes. Elevated DCA levels are associated with worse metabolic and inflammatory profiles and may contribute to disease risk, including cancer development [35]. In the TNBC sub-cohort, higher levels of the conjugated DCA form taurodeoxycholic acid (TDCA) was observed in responders, whereas a higher DCA/TDCA ratio was detected in non-responders further supports that microbial bile acid metabolism may influence clinical outcomes.

We observed specific associations between short-chain fatty acids (SCFAs) and microbial metabolic pathways, highlighting potential mechanistic links between microbial metabolism and host–microbial interactions. Acetate (C2), propionate (C3), and butyrate (C4) were positively correlated with GLUCOSE1PMETAB-PWY, representing glucose and glucose-1-phoshate degradation, suggesting that microbial fermentation of glucose contributes to the production of these SCFAs. In contrast, caproate (C6) exhibited negative correlations with several biosynthetic pathways, including a strong negative association between PWY0-1586, involved in bacterial peptidoglycan synthesis. These findings, however, require validation in functional experiments to confirm the mechanistic relationships.

Our findings identify exploratory associations between gut microbial diversity metrics, specific taxa, and metabolite signatures, and response to neoadjuvant chemotherapy (NAC) in breast cancer. While these results may support the potential use of gut microbiome profiling as a non-invasive biomarker for predicting response to NAC, they should be regarded as hypothesis-generating. Validation in larger, independent cohorts is required to determine if microbiome biomarkers have potential relevance for patient stratification and personalized treatment approaches.

This study has several limitations. The small sample size limits statistical power and increases the risk of false-negative findings. To overcome this limitation, non-parametric tests and permutation-based methods were used for evaluation of statistical significance of findings. In addition, cohort heterogeneity with respect to molecular subtypes and neoadjuvant chemotherapy regimens represent an important potential confounder that may have influenced the observed microbiome and metabolomic profiles. The effect of residual confounding factors, such as diet and unmeasured lifestyle variables, also cannot be excluded and should be addressed in further research. Moreover, functional validation is needed to establish causality between microbiome–metabolite profiles and NAC response.

## 5. Conclusions

Baseline gut microbiome composition and microbial metabolites are associated with response to neoadjuvant chemotherapy in breast cancer. Higher diversity, specific taxa, and short-chain fatty acid and bile acid profiles were associated with pathological complete response. Despite limited size, exploratory trends in the TNBC sub-cohort support potential biological relevance. Larger studies with functional validation are needed to confirm these findings and evaluate the microbiome and its produced metabolites as a non-invasive predictive biomarker.

## Figures and Tables

**Table 1:** The therapy outcome (pCR) of the study group based on tumor molecular subtype and therapy regiment.

Molecular Subtype	Therapy Regimen *	pCR/*n* **
HER2+	A + T + Tar	0/1
	T + P + Tar	4/6
Luminal A	T + Tar	0/1
	T + P + Tar	2/3
Luminal B (HER2−)	A + T	0/6
	A	0/1
Luminal B (HER2+)	T + P + Tar	2/5
	A + Tar	0/1
TNBC	T	1/1
	T + P + IO	0/1
	A + IO	2/2
	A + P + IO	1/1
	A + T	0/3
	A + T + P	4/6
	A + T + P + IO	1/1

* ‘T’—taxanes, ‘A’—anthracyclines, ‘P’—platinum agents, ‘Tar’—targeted agents, ‘IO’—pembrolizumab; ** pCR—number of patients achieving pCR, n—total number of patients.

**Table 2:** Clinical characteristics of study groups stratified by pathological complete response (pCR).

Characteristic	pCR (*n* = 17), *n* (%)	No pCR (*n* = 22), *n* (%)	Statistic	*p* Value	Effect Size	Overall (*n* = 39), *n* (%)
Age, years, median (range) ^a^	50.0 (34.0–65.0)	52.5 (30.0–80.0)	168	0.600	−0.102	51.0 (30.0–80.0)
Age group ^d^			0.010	0.921	0.017	
Over 50	9 (23.1%)	12 (30.8%)				21 (53.8%)
Under 50	8 (20.5%)	10 (25.6%)				18 (46.2%)
Body surface area (bsa), m^2^, mean (SD) ^b^	1.87 (0.15)	1.84 (0.18)	0.579	0.566	−0.187	1.86 (0.17)
Overall clinical stage *^,a^			154	0.810	−0.046	
I	2 (5.6%)	4 (11.1%)				6 (16.7%)
II	9 (25.0%)	6 (16.7%)				15 (41.7%)
III	6 (16.7%)	9 (25.0%)				15 (41.7%)
Clinical T stage ^a^			180	0.846	0.037	
1	6 (15.4%)	8 (20.5%)				14 (35.9%)
2	6 (15.4%)	9 (23.1%)				15 (38.5%)
3	3 (7.7%)	2 (5.1%)				5 (12.8%)
4	2 (5.1%)	3 (7.7%)				5 (12.8%)
Clinical N stage ^a^			179	0.824	−0.043	
0	7 (17.9%)	9 (20.5%)				15 (38.5%)
1	5 (12.8%)	6 (15.4%)				11 (28.2%)
2	1 (2.6%)	4 (10.3%)				5 (12.8%)
3	4 (10.3%)	4 (10.3%)				8 (20.5%)
HR status **^,c^			-	0.056	-	
Negative	12 (30.8%)	8 (20.5%)				20 (51.3%)
Positive	4 (10.3%)	13 (33.3%)				17 (43.6%)
Low-positive	1 (2.6%)	1 (2.6%)				2 (5.1%)
HER2 status			180	0.847	0.037	
Negative	2 (5.1%)	4 (10.3%)				6 (15.4%)
Positive (1+)	7 (17.9%)	4 (10.3%)				11 (28.2%)
Positive (2+)	0 (0.0%)	7 (17.9%)				7 (17.9%)
Positive (3+)	8 (20.5%)	7 (17.9%)				15 (38.5%)
Grade ^d^			2.84	0.092	0.270	
2	7 (17.9%)	15 (38.5%)				22 (56.4%)
3	10 (25.6%)	7 (17.9%)				17 (43.6%)
Germline PV ^c^	5 (12.8%)	0 (0.0%)	-	0.011	-	5 (12.8%)
Ki-67, %, median (range) ^a^	40 (12–80)	30 (4–80)	147	0.256	0.217	35 (4–80)

* Based on AJCC clinical anatomic staging. ** HR status considered positive if ER or PR ≥ 10%, low-positive if ER or PR is between 0 and 10%, otherwise—negative. ^a^ Mann–Whitney test, ^b^ Student’s *t*-test, ^c^ Fisher exact test, ^d^ Chi-square test. - not applicable.

**Table 3:** Differentially abundant genera associated with pathological complete response (pCR) identified by ANCOM-BC2.

Taxon	LFC (pCR vs. No pCR) ^1^	SE ^2^	Statistic	*p* Value	*q* Value ^3^
*g__GGB9089*	3.06	0.31	9.74	2.55 × 10^−5^	0.005
*g__GGB101072*	2.36	0.36	6.54	4.19 × 10^−5^	0.005
*g__GGB42638*	2.12	0.27	7.97	4.49 × 10^−5^	0.005
*g__GGB9511*	2.88	0.30	9.46	7.96 × 10^−5^	0.006
*g__GGB45620*	1.94	0.25	7.77	1.09 × 10^−4^	0.007
*g__Rothia*	−2.14	0.26	−8.12	1.87 × 10^−4^	0.009
*g__GGB3649*	−1.80	0.29	−6.19	1.61 × 10^−4^	0.009
*g__GGB51511*	−1.86	0.32	−5.78	2.67 × 10^−4^	0.011
*g__Leuconostoc*	−2.54	0.40	−6.34	3.88 × 10^−4^	0.014
*g__GGB3288*	1.81	0.32	5.74	4.34 × 10^−4^	0.014
*g__GGB9412*	−2.40	0.46	−5.26	5.23 × 10^−4^	0.014
*g__GGB2977*	−1.59	0.36	−4.42	4.92 × 10^−4^	0.014
*g__Klebsiella*	−2.16	0.34	−6.29	7.49 × 10^−4^	0.018
*g__GGB9365*	−1.83	0.40	−4.56	8.14 × 10^−4^	0.019
*g__GGB32726*	2.23	0.37	6.00	9.62 × 10^−4^	0.020
*g__Dialister*	2.09	0.46	4.57	1.03 × 10^−3^	0.021
*g__GGB9766*	−1.24	0.33	−3.82	1.36 × 10^−3^	0.022
*g__GGB4750*	1.47	0.30	4.84	1.29 × 10^−3^	0.022
*g__GGB3588*	−1.42	0.33	−4.24	1.40 × 10^−3^	0.022
*g__Jingyaoa*	−1.26	0.28	−4.41	1.31 × 10^−3^	0.022
*g__GGB51647*	1.38	0.28	4.93	1.70 × 10^−3^	0.025
*g__Kineothrix*	1.91	0.52	3.68	1.72 × 10^−3^	0.025
*g__GGB9590*	−1.14	0.26	−4.31	1.95 × 10^−3^	0.026
*g__Cuneatibacter*	−1.45	0.28	−5.24	1.93 × 10^−3^	0.026
*g__Youxingia*	−1.21	0.33	−3.64	2.23 × 10^−3^	0.028
*g__Jutongia*	1.51	0.33	4.61	2.46 × 10^−3^	0.030
*g__GGB3351*	1.22	0.29	4.25	2.81 × 10^−3^	0.032
*g__Bittarella_ex_Durand_et_al_2017*	−1.08	0.29	−3.76	2.72 × 10^−3^	0.032
*g__GGB9237*	1.57	0.43	3.68	3.61 × 10^−3^	0.040
*g__GGB4552*	−1.30	0.39	−3.36	3.99 × 10^−3^	0.042
*g__GGB9301*	1.33	0.35	3.81	4.14 × 10^−3^	0.042
*g__GGB3612*	1.35	0.40	3.37	4.17 × 10^−3^	0.042
*g__GGB9186*	−1.04	0.29	−3.54	4.64 × 10^−3^	0.045

^1^ Only features with an absolute log2 fold change > 1 and adjusted *p* value (*q*) < 0.05 are displayed. Positive LFC indicates higher abundance in patients achieving pCR. ^2^ SE standard error of LFC. ^3^ *p* values were adjusted for multiple testing using the Benjamini–Hochberg false discovery rate.

**Table 4:** Significant correlations between SCFAs and microbial metabolic pathways.

SCFA ^1^	Pathway ID	*ϱ* (Spearman)	*p* Value ^2^	FDR	Direction
C2	GLUCOSE1PMETAB-PWY: glucose and glucose-1-phosphate degradation	0.52	0.002	0.20	Positive
C3	GLUCOSE1PMETAB-PWY	0.57	0.001	0.17	Positive
C4	GLUCOSE1PMETAB-PWY	0.51	0.002	0.20	Positive
C6	ASPASN-PWY: superpathway of L-aspartate and L-asparagine biosynthesis	−0.58	<0.001	0.17	Negative
C6	PHOSLIPSYN-PWY: superpathway of phospholipid biosynthesis I (bacteria)	−0.53	0.002	0.20	Negative
C6	PWY-5973: cis-vaccenate biosynthesis	−0.50	0.003	0.20	Negative
C6	PWY-6147: 6-hydroxymethyl-dihydropterin diphosphate biosynthesis I	−0.50	0.003	0.20	Negative
C6	PWY-6897: thiamine diphosphate salvage II	−0.53	0.001	0.20	Negative
C6	PWY-7663: gondoate biosynthesis (anaerobic)	−0.53	0.002	0.20	Negative
C6	PWY0-1586: peptidoglycan maturation (meso-diaminopimelate containing)	−0.72	<0.001	0.004	Negative
C6	PWY4FS-7 and PWY4Fs-8: phosphatidylglycerol biosynthesis I and II (plastidic)	−0.50	0.003	0.20	Negative
C6	THISYN-PWY: superpathway of thiamine diphosphate biosynthesis I	−0.53	0.002	0.20	Negative

^1^ Only features with an absolute *ϱ* > 0.5 and *p* value < 0.05 are displayed. SCFA abbreviations: C2—acetate, C3—propionate, C4—butyrate, C6—caproate. ^2^ *p* values were adjusted for multiple testing using the Benjamini–Hochberg false discovery rate.

**Table 5:** Clinical characteristics of TNBC study groups stratified by pathological complete response (pCR).

Characteristic	pCR (*n* = 9) *n* (%)	No pCR (*n* = 6) *n* (%)	Statistic	*p* Value	Effect Size	Overall (*n* = 15) *n* (%)
Age, years, median (range) ^a^	53.0 (34–64)	57.5 (34–80)	20.5	0.479	−0.241	54.0 (34.0–80.0)
Age group ^c^			-	1.000	-	
Over 50	6 (40.0%)	4 (26.7%)				10 (66.7%)
Under 50	3 (20.0%)	2 (13.3%)				5 (33.3%)
Body surface area (bsa), m^2^, median (range) ^b^	1.92 (1.67–2.10)	1.84 (1.63–2.28)	24.0	0.768	0.111	1.92 (0.17)
Overall clinical stage *^,a^			25.0	0.846	−0.074	
I	2 (13.3%)	2 (13.3%)				4 (26.7%)
II	6 (40.0%)	2 (13.3%)				8 (53.3%)
III	1 (6.7%)	2 (13.3%)				3 (20.0%)
Clinical T stage ^a^			20.5	0.440	0.241	
1	3 (20.0%)	4 (26.7%)				7 (46.7%)
2	5 (33.3%)	1 (6.7%)				6 (40.0%)
3	1 (6.7%)	0 (0.0%)				1 (6.7%)
4	0 (0.0%)	1 (6.7%)				1 (6.7%)
Clinical N stage ^a^			23.5	0.649	0.130	
0	6 (40.0%)	5 (33.3%)				11 (73.3%)
1	3 (13.3%)	0 (0.0%)				2 (13.3%)
2	0 (0.0%)	0 (0.0%)				0 (0.0%)
3	1 (6.7%)	1 (6.7%)				2 (13.3%)
Grade ^c^			-	0.235	-	
2	1 (6.7%)	3 (20.0%)				4 (26.7%)
3	8 (53.3%)	3 (20.0%)				11 (73.3%)
Germline PV ^c^	3 (20.0%)	0 (0.0%)	-	0.229	-	3 (20.0%)
Ki-67, %, median (range) ^a^	60 (25–80)	40 (4–80)	18.5	0.341	0.315	60 (4–80)

* Based on AJCC clinical anatomic staging. ^a^ Mann–-Whitney test, ^b^ Student’s *t*-test, ^c^ Fisher exact test. - not applicable.

**Table 6:** Differentially abundant taxa associated with pathological complete response (pCR) identified by ANCOM-BC2.

Taxon	LFC (pCR vs. No pCR) ^1^	SE ^2^	Statistic	*p* Value	q (*p*.adj.) Value ^3^
*s__Ellagibacter_isourolithinifaciens*	−1.98	0.36	−5.51	5.68 × 10^−4^	0.049
*s__Bacteroides_caccae*	2.34	0.41	5.70	4.53 × 10^−4^	0.049
*s__GGB3321_SGB4394*	−4.61	0.59	−7.80	5.56 × 10^−4^	0.049
*s__GGB9347_SGB14313*	4.12	0.41	10.09	5.42 × 10^−4^	0.049
*s__Clostridium_saudiense*	−3.08	0.27	−11.49	3.28 × 10^−4^	0.049
*s__Brotocaccenecus_cirricatena*	−5.01	0.37	−13.37	1.81 × 10^−4^	0.049
*s__GGB3626_SGB4905*	−3.42	0.29	−11.86	2.90 × 10^−4^	0.049

^1^ Only features with an absolute log2 fold change > 1 and adjusted *p* value (*q*) < 0.05 are displayed. Positive LFC indicates higher abundance in patients achieving pCR. ^2^ SE standard error of LFC. ^3^ *p* values were adjusted for multiple testing using the Benjamini–Hochberg false discovery rate.

## Data Availability

The datasets generated and analyzed during the present study are available from the corresponding author upon reasonable request.

## Abbreviations

The following abbreviations are used in this manuscript:

SCFA	Short-chain fatty acid
BA	Bile-acid
NAC	Neoadjuvant chemotherapy
C2	Acetate
C3	Propionate
C4, iC4	Butyrate, isobutyrate
C5, iC5	Pentanoate, isopentanoate
C6, iC6	Caproate, isocaproate
CA	Cholic acid
CDCA	Chenodeoxycholic acid
DCA	Deoxycholic acid
GCA	Glycocholic acid
GCDCA	Glycochenodeoxycholic acid
GDCA	Glycodeoxycholic acid
GLCA	Glycolithocholic acid
LCA	Litocholic acid
MCA	Muricholic acid
TCA	Taurocholic acid
TCDCA	Taurochenodeoxycholic acid
TDCA	Taurodeoxycholic acid
TLCA	Taurolitocholic acid
TUDCA	Tauroursodeoxycholic acid
UDCA	Ursodeoxycholic acid
PV	Pathogenic variants
pCR	Pathological complete response
TNBC	Triple-negative breast cancer
HR	Hormone receptor
HER2	Human epidermal growth factor receptor 2
PFS	Progression-free survival
OS	Overall survival

## Appendix A

**Table A1:** Significant correlations between BAs and microbial metabolic pathways.

SCFA ^1^	Pathway ID	ϱ (Spearman)	*p* Value ^2^	FDR	Direction
TDCA	PWY-5695: inosine 5′-phosphate degradation	0.51	0.001	0.52	Positive
UDCA	PWY-8187: L-arginine degradation XIII (reductive Stickland reaction)	0.56	<0.001	0.49	Positive
	PWY0-1586: peptidoglycan maturation (meso-diaminopimelate containing)	−0.51	0.001	0.52	Negative
	VALSYN-PWY: L-valine biosynthesis	−0.51	0.001	0.52	Negative

^1^ Only features with an absolute ϱ > 0.5 and *p* value < 0.05 are displayed. BA abbreviations: TDCA—taurodeoxycholic acid, UDCA—ursodeoxycholic acid. ^2^ *p* values were adjusted for multiple testing using the Benjamini–Hochberg false discovery rate.
